# Value of a cure for sickle cell disease in reducing economic disparities

**DOI:** 10.1002/ajh.26617

**Published:** 2022-06-06

**Authors:** Marlon Graf, Rifat Tuly, Meghan Gallagher, Jeff Sullivan, Anupam Bapu Jena

**Affiliations:** ^1^ PRECISIONheor Bethesda Maryland USA; ^2^ Bluebird Bio, Inc. Cambridge Massachusetts USA; ^3^ Harvard Medical School Boston Massachusetts USA

To the Editor:

Sickle cell disease (SCD)* is one of the most common inherited blood disorders in the United States (US), found in nearly 1/3000 Americans^1^ and disproportionately affecting Black (1/365 births) and Hispanic (1/16300) populations.^1^

Clinical manifestations start in early childhood, including severe episodic and chronic pain, multisystem organ failure, stroke, and life‐threatening infections. In addition, patients experience lower academic achievement compared to healthy individuals with similar demographic characteristics, have lower chances of meaningful employment in adulthood, face trouble in attention and concentration, score lower on measures of intelligence,^2^ have increased school absences, and are more likely to repeat grades.^3^ For children with SCD in households below the federal poverty line (FPL) or with parents without higher education, disease impacts are exacerbated.

While newborn screening programs, penicillin prophylaxis and modest improvements in treatment options have enabled individuals with SCD to live into adulthood, current life expectancy (LE) estimates vary but are not beyond the fifth decade.^4^

SCD also perpetuates existing disparities in health and wealth between Blacks, other minority groups, and non‐Hispanic Whites. In an environment where Black Americans disproportionately experience poorer health, educational, and economic outcomes compared to other groups, Black Americans with SCD and their families are left further behind. A recent report by the National Academy of Sciences, Engineering, and Medicine reviewed research on economic disparities, finding higher unemployment rates attributable to lower educational attainment, cognitive impairment, and high treatment burden for those living with SCD, compared to matched controls.^2^ Additionally, children with SCD reported delays in accessing care, a reality consistent across the entire SCD population.

Despite all this, the economic impact of SCD for Black Americans remains poorly understood. A previous cohort simulation model estimated projected lifetime income for individuals with SCD to be $695 000 less than that of matched individuals without SCD, due to differences in LE alone.^5^ However, no studies to date have considered or modeled new earnings trajectories of SCD patients if cured from disease or assessed how changes in earnings would reduce income disparities,† both of which seem meaningful and timely in anticipation of new curative‐intent treatments in this setting.

To quantify effects of a cure on annual income and future earnings, we use a cohort‐based microsimulation model to project lifetime earnings trajectories at different points along the life course among individuals with imputed SCD, pre‐ and post‐cure. Additional detail on the study methodology is available in the Supporting Information S1 to this article.

In our model, a cured individual experienced increased productivity and a new earnings trajectory, due to absence of SCD‐related health crises and hospitalizations along with increased LE and increased opportunity to pursue education.

Among a nationally representative sample of 6352 weighted individuals (SCD: 3176, Non‐SCD: 3176) from the 1997 Child Development Supplement to the Panel Survey on Income Dynamics, we found substantial gaps in earnings between individuals with SCD and comparable individuals without SCD. As shown in Figure 1A, individuals with SCD earn between 42% and 46% less annually than the healthy comparison group. NPV of lifetime earnings of the SCD cohort is 59%–66% lower, and undiscounted lifetime income is 69%–75% lower, compared to matched controls.

**FIGURE 1:**
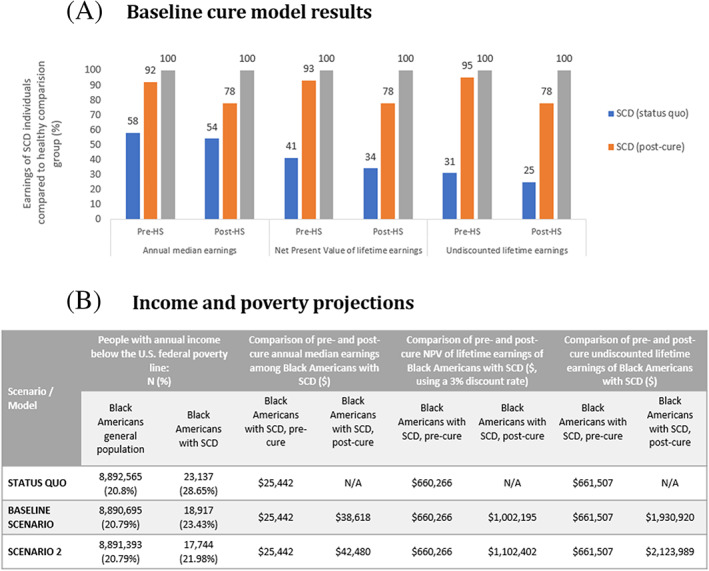
**(A) Baseline curve model results.** Pre‐HS denotes cure model results for individuals receiving treatment before high school, where educational and career pathways may not yet be fully formed. Post‐HS denotes cure model results for individuals receiving treatment during or after high school, where educational and career pathways are likely to be at least partially formed. **(B) Income and poverty projections.** Sources used for calculations: Reported share of Black Americans with income below the federal poverty line (general population)—U.S. Census Bureau, 2020. Count of Black Americans living with SCD—Hassel et al. (2010). Estimated share of Black Americans with income below the federal poverty line (SCD population)—Boulet et al. (2010) and U.S. Census Bureau, 2020. Post‐Cure share of Black Americans with income below the federal poverty line (SCD population)—SCD Trajectory Model Results. Reported annual median income of Black Americans (general population)—U.S. Census Bureau, 2020. Pre‐ and Post‐Cure annual median income of Black Americans)—SCD Trajectory Model Results Key assumptions for calculations of lifetime earnings: Productivity lifespan was 50 years for healthy individuals (18–68), and 26 years for individuals with SCD (18–44)

Introducing a cure partially closes this SCD earnings gap, though some issues may remain due to disease‐related morbidity. For individuals cured before high school, annual earnings are projected to be only 8% lower than those of the healthy comparison group, equaling a 58.6% increase in annual earnings from pre‐ to post‐cure. Additionally, NPV of lifetime earnings and undiscounted lifetime income are projected to be 7% and 5% lower than those of non‐SCD individuals, translating into increases of 126.8% and 206.5%, respectively. Similarly, for individuals receiving the cure during/after high school, when educational and career pathways are likely at least partially formed, annual earnings are projected to be 22% lower than those of the healthy comparison group, while NPV of lifetime earnings and undiscounted lifetime income are both projected to be 22% lower than those of non‐SCD individuals. This means following cure, annual earnings are projected to increase by 44.4%, while NPV of lifetime earnings and undiscounted lifetime income are projected to increase by 136.4% and 212.0%, respectively (Figure 1A).

Additional sensitivity analyses are presented in the Supporting Information S1, but results were directionally consistent across model scenarios.

Model results were then applied to projected income and poverty effects of curing SCD for Black Americans specifically, as they make up the predominant share of the US SCD population.

If a cure were applied today across the Black American population with SCD, annual median income is projected to increase from $25 442 to $38 618 compared to the $45 438 median income of the general Black Americans population. This increase in annual earnings could have substantial effects on poverty rates among a marginalized segment of the US population. The share of Black Americans with SCD earning annual income below FPL would decline from 28.6% (*n* = 23 137) to 23.4% (*n* = 18 917), moving the SCD population closer to the 20.8% share of the overall Black American population in poverty. While not an end goal, this reflects a meaningful reduction (~20%) in earning inequities between individuals with SCD and matched controls. Over a lifetime, effects of curing SCD are even more pronounced: post‐cure, NPV of lifetime earnings of Black Americans with SCD is projected to increase by $341 929 (from $660 266 to $1 002 195, compared to $1 179 172 for Black Americans generally), while undiscounted lifetime earnings would increase by $1 269 413 (from $661 507 to $1 930 920, compared to $2271900) (Figure 1B).

Racial disparities in health, education and wealth are endemic to the United States and are exacerbated for minority groups with severe conditions such as SCD. While our study focused on disparities of Blacks with SCD compared to Black America, we acknowledge equal disparity is by no means the goal. Curing SCD to support economic transformation of persons affected is one of many opportunities to reduce disparities within the Black population, thereby facilitating a more equitable society across demographic lines.

As modeled, a cure would offer immediate improvement in health with downstream improvements in the ability for children to pursue an education similar to their peers, yielding lifelong income benefits. It is also possible benefits of a cure may have generational impact: Lifelong benefits for an individual may help future generations close income gaps, although this has not been formally explored here.

This study focused on quantifying economic benefits of a cure on annual income and future earnings of individuals with SCD. Our study confirmed what is already known: individuals with SCD have lower income trajectories compared to similar individuals without SCD, lower annual earnings, and lower lifetime earnings. These differences are due not only to fewer earning years,^5^ but are very likely the result of disease impact on education and employment preferences and pathways.^1^, ^6^ Our study further shows curing SCD significantly reduces income inequality, leading to greater individual earnings and by extension, societal spillover benefits including diminished reliance on government programs.

Our results expand upon Lubeck et al., who explored associations between SCD and productivity from a lifetime societal perspective and showed projected lifetime income for individuals with SCD was $1 227 000 vs. $1 922 000 for matched individuals without SCD.^5^ This $695 000 income loss was attributed to the 22‐year difference in LE between cohorts.^5^ Our study is directionally consistent with this study, yet offers important extensions such as modeling annual earnings trajectories and career pathways specific to curing SCD versus prolonged disease management.

To our knowledge, our study is the first to model SCD patients to new trajectories following cure, adding important context on the value of innovative therapies for broader societal disparities. The full value of a cure for SCD is not limited to therapeutic effect. Instead, evaluating benefits of a potential cure requires consideration of elements beyond traditional efficacy and productivity, such as spillover effect on education and earnings and resultant impact on equity.

Using SCD as an example, this study illustrates important potential for curative therapies to not only clinically‐transform lives, but to offer broader, profound opportunities for patients and affected communities. Given our model only measures economic benefits related to annual and lifetime incomes and not other aspects such as societal benefits and caregiver burden, benefit of potential cures is likely underestimated. Developing novel curative therapies for SCD and enabling broad access offers significant opportunity to patients and opens‐up meaningful and transformative pathways to reduce inequities tied to health and wealth.

## AUTHOR CONTRIBUTIONS

Marlon Graf, Jeff Sullivan, Anupam Bapu Jena, and Meghan Gallagher conceptualized the study. Marlon Graf, Rifat Tuly, and Jeff Sullivan built the model and analyzed the data, and all authors interpreted the data. Marlon Graf and Rifat Tuly wrote the initial draft of the manuscript, and all authors reviewed and approved the final manuscript.

## FUNDING INFORMATION

Financial support for this study was provided by Bluebird Bio, Inc.

## CONFLICT OF INTEREST

At the time of this study Marlon Graf, Rifat Tuly and Jeff Sullivan were employees of PRECISIONheor, a research consultancy to the health and life sciences industries. Meghan Gallagher is an employee of Bluebird Bio, Inc. and Anupam Bapu Jena is a Professor at Harvard Medical School, and a consultant for PRECISIONheor.

## Supporting information


**Appendix S1** Supporting Information

## Data Availability

The datasets generated and/or analyzed during the current study are available from the corresponding author on reasonable request.

## Endnotes

* For this study, individuals with SCD were identified as children in the National Health Interview Survey (NHIS) whose parental guardian confirmed that “*a doctor or other health professional has ever told the parents their child has sickle cell anemia*.”

† Throughout this paper, *cure* is used to describe hypothetical future genetic therapy; *no cure* is used to describe currently available therapies. The authors acknowledge there is no universal definition of a cure, and that “cure” may mean something different to everyone. For purposes of this model, cure is defined as stoppage of the active manifestation of disease, and based on age of treatment, a potential offsetting of the downstream impact on lifelong disease‐related comorbidity.
