# Supplementary material for: Value of a cure for sickle cell disease in reducing economic disparities
Source: Am J Hematol. 2022 Jun 6;97(8):E289–91. doi: 10.1002/ajh.26617 (PMC9544336; doi:10.1002/ajh.26617)
Supplement: Supplementary file 1 — Appendix S1 Supporting Information [file AJH-97-E289-s001.docx]

**Title:**

Value of a cure for sickle cell disease in reducing economic disparities

**Authors:**

Marlon Graf,^1^ Rifat Tuly,^1^ Meghan Gallagher,^2^ Jeff Sullivan,^1^ Anupam Bapu Jena^3^

**Affiliations:**

1. PRECISIONheor, Los Angeles, CA; 2. bluebird bio, Inc., Cambridge, MA; 3. Harvard Medical School, Boston, MA

**Corresponding author:**

Marlon Graf,

PRECISIONheor,11100 Santa Monica Blvd., Suite 500

310-984-7712

[marlon.graf@precisionvh.com](mailto:marlon.graf@precisionvh.com)

**Supplementary Appendix**

To evaluate economic impacts of curing SCD for a predominantly Black and Hispanic American population, given a hypothetical curative intent therapy, we developed a cohort-based microsimulation model to project lifetime earnings trajectories at different points along the life course among individuals with SCD, pre- and post-cure. A detailed description of the study methodology and model steps is presented below:

*Model Development*

We constructed a cohort simulation model to quantify effects of a curative SCD treatment on annual income and future earnings of individuals with SCD. In our model, a cured individual experienced increased productivity and a new earnings trajectory, due to absence of SCD-related health crises and hospitalizations along with increased life expectancy.

We used data from the Sample Child Core questionnaire of the 2006-2015 National Health Interview Survey (NHIS), an annual, cross-sectional household survey of the U.S. population^1^. The child questionnaire asks a parent or legal guardian whether a doctor or other health professional has ever told them their child has sickle cell anemia^2^. If adults answered yes, children were flagged as having SCD.

Children aged 0-17 were then segmented into SCD and non-SCD cohorts, composed of children who had a dissenting response to the same question. We then estimated individual likelihood of SCD through a logistic regression model, using control variables previously identified as highly correlated with SCD status in NHIS: race/ethnicity; sex; geographic region; parental education; and total family income^2^. Health-related covariates were considered, but ultimately excluded due to endogeneity with SCD diagnosis.

While NHIS has rich information on health conditions and wellbeing among the U.S. population, its cross-sectional nature inhibits analysis of individual health and income trajectories over the life course. As the overarching objective of this study was to estimate impact of a cure for SCD on annual and lifetime earnings, we combined SCD likelihood estimates from NHIS with a longitudinal panel data source, the Panel Study of Income Dynamics (PSID). PSID is a longitudinal biennial survey of economic and demographic behavior such as employment, income, participation in government programs and a range of other measures including health. In 1997, 2002, and 2007, PSID was supplemented with additional data on 0–12-year-olds and their parents through the Child Development Supplement (CDS). Once CDS children turned 18 and completed high school, they moved into the Transition to Adulthood Supplement (TAS) if they were still members of PSID-participating families and then entered the main PSID study once economically independent.

*Study sample*

This panel of PSID children served as our primary analysis cohort, given the strong overlap in age and time period with the NHIS sample used to estimate our SCD likelihood model. We imputed SCD status for everyone in the CDS. First, each respondent’s individual SCD likelihood was calculated using regression coefficients from the NHIS logistic regression model. Second, each observation was split into two observations, where one version was assigned SCD and an individual survey weight ${SvyWeight}_{SCD=1, i,t}={SvyWeight}_{i,t}*{Pr(SCD)}_{i}$and the other version was assigned non-SCD and individual survey weight ${SvyWeight}_{SCD=0, i,t}={SvyWeight}_{i,t}*{[1-\Pr\left( SCD \right))}_{i}]$.

Following imputation of SCD status for the starting CDS cohort, we assumed SCD remained constant throughout each individual’s life. A total of 6,352 weighted patients (SCD: 3,176, Non-SCD: 3,176) from the 1997 CDS were included in the study. *Table 1* describes weighted characteristics of Wave 1 participants in the 1997 Child Development Supplement (CDS), split by SCD status.

*Analysis steps*

At the start of our modeled trajectory, members of the original CDS cohort with imputed SCD status were aged 18-30. Therefore, we used the full PSID adult sample to estimate lifetime earnings trajectories as a function of educational attainment, region, age, gender, and health status. We first estimated earnings trajectories over time for adult respondents in the PSID through a random-effects OLS panel regression, between 2006 and 2015. The outcome of this regression model was an individual’s annual earnings. We controlled for year-FE and clustered errors by individuals and estimated earnings for individual *i* at time *t* through a combination of socio-economic, demographic, and health covariates:

$$\ln\left( {earnings}_{it} \right)=\beta_{0}+\beta_{1}\left( {Socioecon}_{it} \right)+\beta_{2}\left( {Demographic}_{it} \right)+\beta_{3}\left( {Health}_{it} \right)+\partial_{t}+\theta_{i}+\epsilon_{it}$$

Next, we applied coefficients obtained through this regression to individuals from the initial CDS starting cohort, beginning in 2015. Development of earnings and labor market trajectories beyond the observed period was guided by a combination of demographic characteristics from the PSID children’s panel and the trajectories informed by adult PSID respondents. The productivity lifespan for individuals with SCD was truncated at ages 42 for men and 48 for women, congruent with life expectancy estimates^3^. Conversely, average retirement age for healthy individuals was set to 68^4-7^.

Using annual projected earnings for SCD and non-SCD individuals, we then calculated both respondents’ undiscounted lifetime earnings and 2017 net present value (NPV) of lifetime earnings with a 3% discount rate, consistent with best practices recommended by the Second Panel of Cost-Effectiveness in Medicine^8^.

To obtain a comparison of lifetime productivity between individuals who are predicted to have SCD, and a matched comparison group of non-SCD individuals, we then calculated differences between average NPVs of both cohorts.

For undiscounted lifetime earnings, we followed the same calculation steps, but did not discount future earnings.

To estimate the impact of a cure on productivity outcomes of individuals with SCD, we split the treatment population into those who have entered high school (adolescent group, approximated by age ≥14), and those who have not yet reached high school age (children group, age <14). Consistent with the literature on educational and income trajectories, we assumed prior to age 14, receiving treatment would allow individuals to catch-up to a non-SCD earnings trajectory,^9, 10^ as the middle-to-high school transition presents a critical stage in the formation of earnings and educational attainment pathways.^11-13^ We closed the earnings gap by applying average earnings from non-SCD individuals in the same age group to children with SCD who received the cure. As educational and earnings trajectories are at least partially formed for adolescents, we only modeled a partial catch-up in earnings by applying the mid-point between adolescents’ SCD earnings trajectories and the earnings trajectories of non-SCD individuals in the same age group. This partial catch-up approximates mechanisms through which earnings could be increased, such as switching from part-time to full-time work, completing education, and gaining independence. Additionally, our approach for modeling earnings and education benefits assumed patients treated earlier would have a better chance of avoiding permanent chronic complication of disease, which could severely limit long-term productivity. In addition to this earnings benefit, we also extended productivity lifespans to non-SCD levels for those receiving the cure, thus incorporating a lifetime benefit of treatment.

*Sensitivity analyses*

We tested the sensitivity of our results to key assumptions around treatment effect and definition of sickle cell disease, but results of these analyses did not differ from base scenarios in directionality or magnitude and were thus omitted. An overview of the full study methodology and conceptual framework was presented previously by Sullivan et al., 2020^14^.

When extending full earnings benefits to individuals who are older than 14 when receiving a curative treatment, the model projects larger aggregate earnings effects than the one assuming a partial catch-up: when the full earnings benefit is extended to individuals older than 14 at the time of cure, their annual earnings are projected to increase by 75.9%, NPV of lifetime earnings is projected to increase by 187.9%, and undiscounted lifetime income is projected to increase by 308.0%.

*Earnings projections*

Lastly, to project population-level effects of our model on poverty and income levels of the wider African-American population, we combined model estimates with nationally representative data from the U.S. Census and estimates from the literature. While our modeled population was drawn from a U.S. nationally representative sample, survey weights ensured the final study sample represented the demographic makeup of the SCD population.

To calculate how many people could be lifted out of poverty following an SCD cure, we first obtained the number of African-Americans living with SCD,^15^ the current share of them living in poverty from the academic literature,^2^ as well as the share of the general African-American population living in poverty.^16^ We then assumed that curing SCD meant both populations had the share of people in poverty, and applied the earnings gap estimates obtained from cure model scenario *s*:

${N\_Pov}_{SCD}^{Post,s}=N_{SCD}^{Pre}* \frac{{Pct\_Earn}_{SCD}^{Post, s}}{{Pct\_Earn}_{SCD}^{Pre}}*({Pct\_Pov}_{SCD}^{Pre}-{Pct\_Pov}_{genpop}^{Pre})$

Similarly, we also estimated persisting earnings differences between cured SCD individuals and the general African-American population,^17^ again assuming that a cure would mean closing the earnings gap. For cure model scenario *s*, we combined earnings data for the general African-American population with both pre- and post-cure estimates of the gap between individuals with SCD and the healthy comparison group:

$${Earn\_Diff}_{s}=({Earn}_{genpop}^{Pre}*\left( 1-{Pct\_Earn}_{SCD}^{Post,s} \right))-({Earn}_{genpop}^{Pre}*\left( 1-{Pct\_Earn}_{SCD}^{Pre} \right))$$

*Limitations*

Studying rare diseases using secondary data presents numerous challenges. For example, we relied on PSID which has a small sample size relative to other data sets^18^, as our estimates required longitudinal data of a specific nature. As PSID does not ask respondents about SCD, we inferred individual likelihood of SCD based on a probabilistic model, using the NHIS. Our probabilistic model of identifying individuals with SCD is based on observable factors that have been shown to be heavily correlated with SCD (health and socioeconomic), we are unable to account for any unobservable characteristics in our imputation process.

Individual earnings trajectories are informed by a regression model of PSID adults as we do not observe actual earnings of individuals in the CDS past ages 18-30 in 2017, the last year of survey data. This is particularly relevant for mid- and late-career earnings pathways as the CDS, to date, only covers childhood, adolescence, and early adulthood.

Our evaluation of the impact of a cure on SCD required several assumptions, including duration of lifetime earnings and likely earnings benefits of a cure. These and other model assumptions directly impact results but were made to explore a realistic scenario given current knowledge. We hypothesize much of the increased earnings are due to improvements in educational attainment^19-21^. However, we do not observe or model educational attainment directly. Furthermore, as our model is limited to economic outcomes only, it does not explicitly account for moderating health outcomes such as regimen-related morbidity and mortality from gene therapy that may curb future earnings potential.

**References**

1. Botman S, Moriarity CL. Design and estimation for the National Health Interview Survey, 1995-2004. Journal Issue. 2000;Vital and health statistics. Series 2, Data evaluation and methods research ; no. 130.

2. Boulet SL, Yanni EA, Creary MS, Olney RS. Health Status and Healthcare Use in a National Sample of Children with Sickle Cell Disease. *American Journal of Preventive Medicine*. 2010;38(4):S528-S535. doi:10.1016/j.amepre.2010.01.003

3. Platt OS, Brambilla DJ, Rosse WF, et al. Mortality In Sickle Cell Disease -- Life Expectancy and Risk Factors for Early Death. *New England Journal of Medicine*. 1994;330(23):1639-1644. doi:10.1056/nejm199406093302303

4. Torpey; MTaE. *Older workers: Labor force trends and career options*. 2017. <https://www.bls.gov/careeroutlook/2017/article/older-workers.htm>

5. Bureau of Labor Statistics USDoL, The Economics Daily;. *Labor force participation rate for workers age 75 and older projected to be over 10 percent by 2026*. <https://www.bls.gov/opub/ted/2019/labor-force-participation-rate-for-workers-age-75-and-older-projected-to-be-over-10-percent-by-2026.htm>

6. Bureau of Labor Statistics USDoL, The Economics Daily;. *Civilian labor force participation rates by age, sex, race, and ethnicity, 1999, 2009, 2019, and projected 2029 (in percent)*. <https://www.bls.gov/emp/tables/civilian-labor-force-participation-rate.htm>

7. Munnell AH. Social security’s real retirement age is 70.

8. Sanders GD, Neumann PJ, Basu A, et al. Recommendations for conduct, methodological practices, and reporting of cost-effectiveness analyses: second panel on cost-effectiveness in health and medicine. *Jama*. 2016;316(10):1093-1103.

9. De Nardi M, Pashchenko S, Porapakkarm P. *The lifetime costs of bad health*. 2017. 0898-2937.

10. Smith JP. The impact of childhood health on adult labor market outcomes. *The review of economics and statistics*. 2009;91(3):478-489.

11. Lin Y, Liu VYT. Timing matters: How delaying college enrollment affects earnings trajectories. 2019;

12. Alspaugh JW. Achievement loss associated with the transition to middle school and high school. *The Journal of educational research*. 1998;92(1):20-25.

13. Bozick R, DeLuca S. Better late than never? Delayed enrollment in the high school to college transition. *Social Forces*. 2005;84(1):531-554.

14. Sullivan J, Graf M, Tuly R, et al. The Impact of Curative Therapy on Socioeconomic Disparities Among Children with Sickle Cell Disease. CELL PRESS 50 HAMPSHIRE ST, FLOOR 5, CAMBRIDGE, MA 02139 USA; 2020:358-359.

15. Hassell KL. Population estimates of sickle cell disease in the US. *American journal of preventive medicine*. 2010;38(4):S512-S521.

16. U.S. Census Bureau. Table B-1. People in Poverty by Selected Characteristics: 2017 and 2018. <https://www2.census.gov/programs-surveys/demo/tables/p60/266/tableB-1.xls>

17. U.S. Census Bureau. Table A-1. Income Summary Measures by Selected Characteristics: 2018 and 2019. <https://www2.census.gov/programs-surveys/demo/tables/p60/270/tableA1.xlsx>

18. Moffitt R, Zhang S. The PSID and Income Volatility: Its Record of Seminal Research and Some New Findings. *Ann Am Acad Pol Soc Sci*. 2018;680(1):48-81. doi:10.1177/0002716218791766

19. Choi AI, Weekley CC, Chen S-C, et al. Association of educational attainment with chronic disease and mortality: the Kidney Early Evaluation Program (KEEP). *Am J Kidney Dis*. 2011;58(2):228-234. doi:10.1053/j.ajkd.2011.02.388

20. Schatz J. Brief Report: Academic Attainment in Children With Sickle Cell Disease. *Journal of Pediatric Psychology*. 2004;29(8):627-633. doi:10.1093/jpepsy/jsh065

21. Tamborini CR, Kim C, Sakamoto A. Education and Lifetime Earnings in the United States. *Demography*. 2015;52(4):1383-1407. doi:10.1007/s13524-015-0407-0

**Additional Tables and Figures**

*Table S1.* *Weighted key characteristics of Wave 1 participants in the 1997 Child Development Supplement (CDS), split by sickle cell disease status*

| **Variable** | **SCD cohort (N=3,176)** | | **Non- SCD cohort (N=3,176)** | |
| --- | --- | --- | --- | --- |
|  | *Mean* | *SD* | *Mean* | *SD* |
| **Age at initial survey wave** | 6.18 | 3.52 | 6.13 | 3.62 |
|  | *Count* | *%* | *Count* | *%* |
| **Gender**  Male  Female | 1,574  1,602 | 49.56%  50.44% | 1,558  1,618 | 49.07%  50.93% |
| **Region**  Northeast  Midwest  South  West | 343  1,103  1,637  93 | 10.80%  34.75%  51.53%  2.92% | 579  862  1,147  588 | 18.23%  27.16%  36.10%  18.51% |
| **Race/ethnicity**  Non-Hispanic White  Black Americans  Hispanic  Other | 171  2,862  18  125 | 5.3%  90.10%  0.57%  3.93% | 2,368  579  88  140 | 74.57%  18.23%  2.77%  4.42% |

**Note:** to impute sickle cell status into CDS, each observation was split into two observations, where one version was assigned SCD and an individual survey weight of $\mathrm{SvyWeight}_{SCD=1, i,t}=\mathrm{SvyWeight}_{i,t}*{Pr(SCD)}_{i}$, and the other version was assigned non-SCD as well as an individual survey weight of $\mathrm{SvyWeight}_{SCD=0, i,t}=\mathrm{SvyWeight}_{i,t}*{[1-\Pr\left( \mathrm{SCD} \right))}_{i}]$.

*Table S2. Cure model results*

| **Population** | **Median Annual Income** | | **NPV of Lifetime Earnings** | | **Lifetime Earnings (undiscounted)** | |
| --- | --- | --- | --- | --- | --- | --- |
|  | **Annual earnings gap % (No Cure)** | **Annual earnings gap %**  **(Cure)** | **Lifetime NPV Gap % (No Cure)** | **Lifetime NPV Gap % (Cure)** | **Lifetime Earnings Gap % (No Cure)** | **Lifetime Earnings Gap % (Cure)** |
| **BASELINE SCENARIO**   - Children <14 receive the full earnings benefit, while anyone >=14 receives partial earnings benefit - Baseline SCD definition = predicted SCD obtained from likelihood model | | | | | | |
| **Children aged 10-13 at cure** | 42% | 8% | 59% | 7% | 69% | 5% |
| **Observations aged 14+ at cure** | 46% | 22% | 67% | 22% | 75% | 22% |
| **SCENARIO 2**   - Everyone receives the full earnings benefit regardless of age - Baseline SCD definition = predicted SCD obtained from likelihood model | | | | | | |
| **Children aged 10-13 at cure** | 42% | 8% | 59% | 7% | 69% | 5% |
| **Observations aged 14+ at cure** | 46% | 5% | 67% | 5% | 75% | -2% |

**Note:** Across model scenarios, cure is used to describe hypothetical future genetic therapy that is universally effective and has no toxicity; no cure is used to describe currently available therapies.

*Table S3. Comparison of income and poverty rates, by race*

| **Population** | **People with annual income below the U.S. federal poverty line:  N (%)** | | | **Annual median earnings for selected population groups ($)** | | | **NPV of lifetime earnings for selected populations ($, using a 3% discount rate)** | | | **Undiscounted lifetime earnings for selected populations ($)** | | |
| --- | --- | --- | --- | --- | --- | --- | --- | --- | --- | --- | --- | --- |
|  | **non-Hisp-White general population** | **non-Hisp-Black general population** | **non-Hisp-Black with SCD** | **non-Hisp-White general population** | **non-Hisp Black general population** | **non-Hisp-Blacks with SCD, post-cure** | **non-Hisp-White general population** | **non-Hisp Black general population** | **non-Hisp-Blacks with SCD, post-cure** | **non-Hisp-White general population** | **non-Hisp Black general population** | **non-Hisp-Blacks with SCD, post-cure** |
| **STATUS QUO** | 15,780,015 (8.1%) | 8,892,565 (20.8%) | 23,137 (28.65%) | $76,057 | $45,438 | N/A | $1,973,772 | $1,179,171 | N/A | 3,878,907 | $2,271,900 | N/A |
| **BASELINE SCENARIO** | 15,780,015 (8.1%) | 8,890,695 (20.79%) | 18,917  (23.43%) | $76,057 | $45,438 | $38,618 | $1,973,772 | $1,179,171 | $1,002,195 | 3,878,907 | $2,271,900 | $1,930,920 |
| **SCENARIO 2** | 15,780,015 (8.1%) | 8,891,393 (20.79%) | 17,744  (21.98%) | $76,057 | $45,438 | $42,480 | $1,973,772 | $1,179,171 | $1,102,402 | 3,878,907 | $2,271,900 | $2,123,989 |

**Sources used for calculations:**

- Reported share of individuals with income below the federal poverty line (general population, by racial group) – [U.S. Census Bureau, 2020](https://www2.census.gov/programs-surveys/demo/tables/p60/266/tableB-1.xls)
- Count of Black Americans living with SCD – [Hassel et al. 2010](https://www.ajpmonline.org/article/S0749-3797(09)00960-X/fulltext)
- Post-Cure share of Black Americans with income below the federal poverty line (SCD population) – SCD Trajectory Model Results
- Reported annual median income of the general population, by race – [U.S. Census Bureau, 2020](https://www2.census.gov/programs-surveys/demo/tables/p60/266/tableB-1.xls)
- Post-Cure annual median income of Black Americans – SCD Trajectory Model Results

**Key assumptions for calculations of lifetime earnings:**

- Productivity lifespan was 50 years for healthy individuals (18-68), and 26 years for individuals with SCD (18-44)

*Figure S1. Comparison of annual median earnings pre- and post-cure, assuming cure does not lead to extended life benefit*
